# Fitness of Outer Membrane Vesicles From *Komagataeibacter intermedius* Is Altered Under the Impact of Simulated Mars-like Stressors Outside the International Space Station

**DOI:** 10.3389/fmicb.2020.01268

**Published:** 2020-06-26

**Authors:** Olga Podolich, Olga Kukharenko, Iryna Zaets, Iryna Orlovska, Larysa Palchykovska, Leonid Zaika, Serhii Sysoliatin, Ganna Zubova, Oleg Reva, Maxym Galkin, Tetyana Horid’ko, Halyna Kosiakova, Tatiana Borisova, Volodymyr Kravchenko, Mykola Skoryk, Maxym Kremenskoy, Preetam Ghosh, Debmalya Barh, Aristóteles Góes-Neto, Vasco Azevedo, Jean-Pierre de Vera, Natalia Kozyrovska

**Affiliations:** ^1^Institute of Molecular Biology and Genetics of NASU, Kyiv, Ukraine; ^2^Centre for Bioinformatics and Computational Biology, University of Pretoria, Pretoria, South Africa; ^3^Palladin Institute of Biochemistry of NASU, Kyiv, Ukraine; ^4^NanoMedTech LLC, Kyiv, Ukraine; ^5^Department of Computer Science, Virginia Commonwealth University, Richmond, VA, United States; ^6^Centre for Genomics and Applied Gene Technology, Institute of Integrative Omics and Applied Biotechnology (IIOAB), Purba Medinipur, India; ^7^Molecular and Computational Biology of Fungi Laboratory, Department of Microbiology, Institute of Biological Sciences, Federal University of Minas Gerais, Belo Horizonte, Brazil; ^8^Laboratory of Cellular and Molecular Genetics, Department of General Biology, Institute of Biological Sciences, Federal University of Minas Gerais, Belo Horizonte, Brazil; ^9^Institute of Planetary Research, German Aerospace Center, Berlin, Germany

**Keywords:** outer membrane vesicles, Mars-like stressors, lipids, functionality, biosafety

## Abstract

Outer membrane vesicles (OMVs), produced by nonpathogenic Gram-negative bacteria, have potentially useful biotechnological applications in extraterrestrial extreme environments. However, their biological effects under the impact of various stressors have to be elucidated for safety reasons. In the spaceflight experiment, model biofilm kombucha microbial community (KMC) samples, in which *Komagataeibacter intermedius* was a dominant community-member, were exposed under simulated Martian factors (i.e., pressure, atmosphere, and UV-illumination) outside the International Space Station (ISS) for 1.5 years. In this study, we have determined that OMVs from post-flight *K. intermedius* displayed changes in membrane composition, depending on the location of the samples and some other factors. Membrane lipids such as sterols, fatty acids (FAs), and phospholipids (PLs) were modulated under the Mars-like stressors, and saturated FAs, as well as both short-chain saturated and trans FAs, appeared in the membranes of OMVs shed by both post-UV-illuminated and “dark” bacteria. The relative content of zwitterionic and anionic PLs changed, producing a change in surface properties of outer membranes, thereby resulting in a loss of interaction capability with polynucleotides. The changed composition of membranes promoted a bigger OMV size, which correlated with changes of OMV fitness. Biochemical characterization of the membrane-associated enzymes revealed an increase in their activity (DNAse, dehydrogenase) compared to wild type. Other functional membrane-associated capabilities of OMVs (e.g., proton accumulation, interaction with linear DNA, or synaptosomes) were also altered after exposure to the spaceflight stressors. Despite alterations in membranes, vesicles did not acquire endotoxicity, cytotoxicity, and neurotoxicity. Altogether, our results show that OMVs, originating from rationally selected nonpathogenic Gram-negative bacteria, can be considered as candidates in the design of postbiotics or edible mucosal vaccines for *in situ* production in extreme environment. Furthermore, these OMVs could also be used as promising delivery vectors for applications in Astromedicine.

## Introduction

Different populations of outer membrane vesicles (OMVs) produced by Gram-negative bacteria are naturally enriched with lipopolysaccharides (LPSs), nucleic acids, lipids, and proteins. Such contents endow vesicles with distinct capabilities for interacting with surrounding biopolymers, bacteriophages, cells, or organisms (Schwechheimer and Kuehn, 2015; Toyofuku et al., 2019). OMVs of Gram-negative bacteria have several functions related to intercellular communications and signaling events (Ahmadi Badi et al., 2017) that include transfer of enzymes and antimicrobials (Schulz et al., 2018), immunomodulation (Cañas et al., 2018; Ahmadi Badi et al., 2019; Ladinsky et al., 2019; Molina-Tijeras et al., 2019), contribution to intestinal homeostasis (Patten et al., 2017; Cecil et al., 2019), and epigenetic processes (Celluzzi and Masotti, 2016; Vdovikova et al., 2018). Multifunctionality of OMVs endow bacteria the capability to withstand changed or harsh environment, and this phenomenon has attracted research interests in elucidating adaptation mechanisms under stress-related conditions. McBroom and Kuehn (2007) showed that vesiculation is an independent stress response that mobilizes bacterial populations for survival *via* alterations in their cell membranes that enhance the export of damaged polymer products under environmental stress.

Spaceflight-associated and simulated Mars-like stressors such as radiation, changed gravity and atmosphere, and temperature fluctuations can be challenging for the survival of microorganisms, and a complex mixture of these extraterrestrial factors promotes global genomic, proteomic, and secondary metabolomic changes that result in impaired cellular processes and functions, affecting cell growth, cell morphology and development, virulence and resistance, and biofilm formation (Huang et al., 2018; Zea et al., 2018; de Vera et al., 2019; Romsdahl et al., 2019). Microorganisms evolve different response and adaptation mechanisms to extreme environments; however, many of them are still unknown. Biological effects of OMVs shed by nonpathogenic Gram-negative bacteria under stressful conditions remain to be elucidated from the point of view of their practical application in extraterrestrial settlements. Under space exploration, microorganisms can produce many special secondary metabolites that could be fabricated (Camere and Karana, 2018) and used as medicine for both humans and animals (e.g., antibiotics, vaccines; Huang et al., 2018). An issue of practical significance for such extracellular vesicles (EVs) is the evaluation of possible risks in terms of safety of modified vesicles with alternated functionality under the effects of various stressors.

A growing body of preclinical and clinical evidence supports the concept of bidirectional microbiota-gut-brain interactions (Mohajeri et al., 2018; Toribio-Mateas, 2018; Mohajeri, 2019), in which OMVs are active players (Choi et al., 2019; Ladinsky et al., 2019). In this context, the maintenance of healthy gut microbiota during the interplanetary journeys, e.g., to Mars, will be a challenge in the near future. During a spaceflight, the lifestyle (diet, low physical activities, and antibiotic usage, etc.) of crew-members, sterility of spaceship confined systems, and environmental stresses (radiation, changed gravity, etc.) result in microbiome alterations, mainly, in species richness, without any significant effects onto the species diversity (Iosim et al., 2019; Voorhies et al., 2019). On the other hand, spaceflight and associated stressors promote changes in gut bacterial physiology (Nickerson et al., 2004; Wilson et al., 2007; Morrison et al., 2019) that could potentially lead to an impaired immunity (Guéguinou et al., 2009; Crucian et al., 2015; Cervantes and Hong, 2016) and predisposition to illnesses among astronauts (Smith, 2018). This concern is heightened by the reported increase in virulence of pathogens in microgravity conditions, as well as a potentially increased risk of bacterial and viral infections (Mehta et al., 2014). Mitigating the adverse effects on astronauts’ health has been difficult and mostly included palliative care, which involves an integrated approach or some medications (Iosim et al., 2019). In the future, during the spaceflight, astronauts may be given cellular therapy for improving their response to spaceflight and helping to ameliorate known hazards (e.g., radiation; Iosim et al., 2019; Schäfer, 2019). While the safety of cell therapy application under extreme conditions is yet to be proven, noncellular EV-based therapy can serve as a safe substitute. The restoration of normal microbial community during a spaceflight may be facilitated by mucosal vaccination, using OMVs of safe rationally selected bacteria or microbiome-targeted probiotics (Sakai et al., 2018; Gorreja, 2019). Protective mucosal immune responses are most effectively induced by mucosal immunization through oral, nasal, rectal, vaginal routes, or by injection (Neutra and Kozlowski, 2006). In spite of significant shifts, occurring in immune function pathways under spaceflight, vaccination is quite possible and T-cell response is expected to be adequate (Iosim et al., 2019). OMVs could also be potentially employed for treating stress-induced depression of crew members during spaceflight (Choi et al., 2019) or amelioration of metabolic dysfunctions (Ashrafian et al., 2019).

In the Biology and Mars Experiment (BIOMEX; de Vera et al., 2019), living kombucha microbial community (KMC; bacterial and yeast species, inhabiting the cellulose-based pellicle film) was exposed outside the International Space Station (ISS) under simulated Mars-like stressors with the purpose of finding out the extent of survival of the KMC-members, as well as to assess the safety of the returned microbial samples and their nanostructures. An increased pool of EVs, produced by the surviving post-flight KMC-members, was discovered and has been published recently (Podolich et al., 2019). The purpose of this study was to characterize the vesicleome of a key KMC bacterium – *Komagataeibacter intermedius* – exposed to space/Mars-like stressors. Additionally, we aimed to assess whether the populations of OMVs shed by the recovered bacterial strains show any changes in their fitness, and whether these alterations compromise in anyway the safety of the *K. intermedius* OMVs compared to the KMC metavesicleome (all the membrane vesicle populations shed by many individual microorganisms, KMC-members).

## Materials and Methods

### Exposure Conditions and Reactivation of Space Returned KMC Samples

Dry organo-mineral samples, containing embedded KMC pellicle fragments, were placed in three positions (top, middle, and bottom) in carrier C2 in tray 2, which has a three-level architecture, each one hosting four kombucha samples (along with samples of other experiments) and maintained under a simulated Mars atmosphere (95.55% CO_2_, 2.70% N_2_, 1.60% Ar, 0.15% O_2_, and ~370 ppm H_2_O) and a pressure of 980 Pa (Supplementary Figure S1). The top level (tKMCs) samples were additionally exposed to a solar UV flux cutoff by optical filters to wavelengths of >200 nm as prevalent on the Martian surface, whereas the middle (mKMC) and bottom (bKMC) levels were kept in darkness (protected from UV radiation); however, in Mars-like atmosphere and pressure for reference. Returned KMC samples were cultivated for reactivation in a filter-sterilized black tea, as previously described (Podolich et al., 2017).

### Culture of Kombucha and Bacterial Strains

Kombucha culture of ecotype IMBG-1 was grown in filter-sterilized sugared (7%) black tea infusions at 28°C, for 3–5 days. The previously re-isolated strains of *K. intermedius* IMBG180 from initial KMC (iKMC), 183 (bKMC), 184 (mKMC), and 185 (tKMC; Podolich et al., 2019) were cultured in HS medium (Hestrin and Schramm, 1954) at 28°C, for 3 days. *Bacillus subtilis* IMBG132, *Escherichia coli* S-17 (obtained from Prof. A. Puehler, Germany), and *Pseudomonas aeruginosa* IMBG188 (the collection of Institute of Molecular Biology and Genetics, Kyiv, Ukraine) were cultured in LB medium (Miller, 1972) at 28°C, overnight for antimicrobial assays. *E. coli* DH5α (pTZ19R; Thermo Fisher Scientific, Lithuania) was grown overnight in LB using ampicillin (100 mg ml^−1^).

### EVs and OMVs Isolation and Visualization

Reactivated kombucha planktonic culture (at the stage when the pellicle appeared) or *K. intermedius* cultures were centrifuged at 17,000 rpm for 20 min at 4°C. The supernatants were collected and further ultra-centrifuged at 100,000 × *g* for 1 h at 4°C (Beckman Instruments Inc., L8M, rotor 55.2 Ti). The resulting pellets were resuspended in sterile filtered (0.10 μ pore size filter; Minisart, Sartorius, Germany) phosphate-buffered saline (PBS). Before visualization, fresh vesicle preparations were filtered (0.20 μ). The isolated vesicle preparations were estimated by the microvolume protein content determination method using NanoDrop ND-1000 spectrophotometer (NanoDrop Technologies, Wilmington, DE). Visualization of isolated vesicles was performed using transmission electron microscopy (TEM) and scanning electron microscopy, and the relative size of vesicles was estimated by dynamic light scattering (DLS), as previously described (Podolich et al., 2019). Zeta potential determination was performed on a Zetasizer Nano S (Malvern, UK) at 25°C. Colloids were irradiated with helium-neon laser with *λ* = 633 nm, and the scattered light was recorded at an angle of 173°.

In this study, we adopted the following scientific community terms: EVs for vesicles produced by KMC-members (bacteria and yeasts) and OMV for Gram-negative bacterium *K. intermedius*.

### DNA and RNA Isolation From OMVs and Analyses

DNAse I (final concentration of 1 μg/μl, Thermo Fisher Scientific, Lithuania) was added to the OMV samples and incubated at 37°C, for 30 min. A 50 mM EDTA solution was used for inactivation of DNAse I at 65°C, for 10 min. Total DNA from *K. intermedius* OMVs was isolated with the PowerSoil DNA Isolation Kit (MO BIO Laboratories, USA).

Total RNA from the OMVs was isolated with an RNA Clean & Concentrator kit (Zymo Research Corp., USA). In separate experiments before RNA extraction, the OMVs were treated with RNase A (final concentration of 1 μg/μl, Thermo Fisher Scientific, Lithuania) for 30 min, at 37°C and frozen for 15 min, at −20°C, to stop RNase before vesicle lysis. RNA integrity was validated by the microchip electrophoresis system (MCE-202/MultiNA SHIMADZU, Japan).

### Lipid Analyses

Total lipids were extracted from vesicle samples as recommended (Bligh and Dyer, 1959; Palmer, 1971). The lipid extracts were separated by thin-layer chromatography on standard plates, using a solvent system of hexane: diethyl ether:glacial acetic acid (85:15:1, by vol.). The total phospholipid (PL) content was determined, as previously described (Vaskovsky et al., 1975). The Carlo Erba HRGC 5300 gas chromatograph (Italy) with flame ionization detector equipped with a glass packed column (length: 3.5 m, internal diameter: 3.0 mm) completed with 10% SP-2300 phase (Silar 5CP) on Chromosorb W/HP was used to separate and identify FA methyl esters. The temperature was programmed from 140 to 250°C, at 2°C/min with a final hold. The Agilent Technologies 7890A gas chromatograph (USA) with SP-2560 column (100 m × 0.25 mm, df 0.20 μm, Germany) was used to separate and identify cis/trans isomers of unsaturated FA methyl esters. The temperature was programmed from 140 to 240°C, at 4°C/min with a final hold. Individual FAs in samples were identified on the basis of their retention time compared to appropriate commercially available standards (Sigma-Aldrich, USA; Serva, Germany). Results were expressed as percentage of total FAs. All the experiments were repeated three times.

### The Electrophoresis Mobility Shift Assay (EMSA)

The interaction of the OMVs with plasmid pTZ19R DNA *Eco*RI-linearized form (Fermentas, Lithuania) was indicated by a slower electrophoretic mobility of their complexes in the agarose gel, as described in Podolich et al. (2019).

### Assessment of the Translocation of Protons Across Membranes of OMVs

The vesicle suspensions (0.1 mg ml^−1^ final protein concentration) in the PBS supplemented with 2 mM CaCl_2_ were pre-incubated for 10 min, at 37°C, and then transferred to a stirred cuvette thermostat at 37°C. The recording was initiated from the time of the addition of acridine orange to the final concentration of 5 μM. Fluorescence changes were measured using a Hitachi MPF-4 spectrofluorometer at the excitation and emission wavelengths of 490 and 530 nm, respectively.

### OMVs Dehydrogenase(s) Activity Assay

Тhe MTT [a tetrazolium salt, 3-(4,5-dimethyl-thiazoyl-2-yl)-2,5-diphenyltetrazolium bromide] assay (Mosmann, 1983) was used with some modifications. In brief, a sample of 0.1 ml of OMVs (4 mg ml^−1^ in protein) in PBS was incubated with 10 μl of 2 mg ml^−1^ MTT solution (in PBS) for 2.5 h, at 37°C. After incubation, the samples were centrifuged at 8,000 × *g* for 2 min. The formazan pellet was dissolved in 10 μl dimethyl sulfoxide (DMSO). Optical absorbance of the converted dye was measured at 570 nm. The molar extinction coefficient for MTT-formazan in DMSO, needed to calculate its content in the samples, was 1.35 × 10^4^ M^−1^ cm^−1^. The results were converted into mM of produced formazan mg^−1^ protein min^−1^.

### Antimicrobial Activity of OMVs and EVs

Antimicrobial effects of both OMVs and KMC EV preparations on model living bacteria were determined by the spot test method, using minimal vesicle concentration that generated clear halos on a bacterial lawn. The grown lawn of target cells (*B. subtilis*, *E. coli*, *P. aeruginosa*) was coated with 20 μl of vesicle preparation (2 mg ml^−1^ of the total protein). The agar plates were incubated at 30°C, overnight. Antimicrobial activity of vesicles was indicated by the appearance of clear zones at the site of vesicle sample application. A sterile PBS was used as a negative control.

### Plasmid DNA Degradation Assay

Plasmid pTZ19R DNA from *E. coli* DH5α was isolated with the innuPREP Plasmid Mini Kit 2.0 (Analytik Jena AG, Germany). OMVs (5 mg ml^−1^) were suspended in PBS (pH 7.4), filtered (0.20 μ), and added to 300 ng of plasmid pTZ19R DNA (Fermentas, Lithuania), and a final reaction mixture (20 ml) was incubated for 16 h at 37°C in the DEPC-treated water. After incubation, the samples were separated by electrophoresis in 1.2% agarose gel in Tris acetate-EDTA buffer (pH 7.0). The stained gels were visualized under the UV-light.

### Murine and Human Cell Lines and Culture Conditions

Murine embryonic fibroblast (MEF) cells were obtained from muscles of the buttocks of a 17-day murine embryo, as described in Jozefczuk et al. (2012) and cultured in DMЕМ-F12 (Biowest, USA) supplemented with the Biowest Antibiotic-Antimycotic solution (2.0 μl/ml). Human colon carcinoma cells COLO 205 (Sigma-Aldrich, USA) were cultured in RPMI 1640 (Sigma-Aldrich, USA) with heat-inactivated fetal bovine serum (10%), 2 mМ ʟ-glutamine, and gentamicin (40 μg/ml) added. Cultures were incubated at 37°C in a humidified atmosphere of 5% CO_2_/95% air in 100 ml flasks.

### Cellular Uptake of Fluorescently Labeled OMVs

Isolated OMVs were labeled with DiO (3,3′-dioctadecyloxacarbocyanine perchlorate; Thermo Fisher Scientific, USA), a fluorescent lipophilic stain, with 20 μg/ml. Nuclei were stained with 0.4 μg/ml DAPI (4′,6-diamidine-2′-phenylindole dihydrochloride; Sigma-Aldrich, USA), and imaging was performed using LEICA DM1000 microscope (100× objective lens).

### Cytotoxicity Assay

MEF or COLO 205 cells were harvested by centrifugation and suspended at lg5 cells/ml in complete medium. Fifty-microliter aliquots of the cell suspension were transferred in duplicate to the 96-well microtiter plate, containing diluted samples (total volume, 100 μl). As a positive control, amitozyn (1 mg ml^−1^; Hermant et al., 2013) kindly provided by Dr. A. Potopalsky (IMBG, Kyiv) was used, and as a negative control, PBS was used. Cytotoxicity test was performed using the MTT reduction assay (Mosmann, 1983). The optical density of each well was measured with a microplate spectrophotometer (Multiscan Tirertek MMC 340, USA) equipped with a 540 nm filter.

### Endotoxicity Assay

Biological activity of OMV-associated LPS was quantified by the end-point chromogenic *Limulus* amebocyte lysate (LAL) assay, using Pierce™ LAL Chromogenic Endotoxin Quantitation Kit (Thermo Fisher Scientific, USA). The optical density of each sample was measured at 405 nm by NanoDrop ND-1000 spectrophotometer (NanoDrop Technologies, USA). Endotoxin concentration in test samples was calculated using the *E. coli* Endotoxin Standard (011:B4) for calibration.

### OMVs Interaction With Synaptosomes

Experiments on neurotoxicity risk assessment have been performed at the neurochemical level of the nervous system organization and involved an analysis of radiolabeled glutamate transport characteristics, which is a key excitatory neurotransmitter in the central nervous system, using synaptosomes (according to Guidelines for Neurotoxicity Risk Assessment of US Environmental Protection Agency, 1998, based on paragraph 3. Hazard Characterization: 3.1.3.4. In Vitro Data in Neurotoxicology; US Environmental Protection Agency, 1998). Synaptosomes’ preparation from a rat brain and measurements of the ambient level of ʟ-[^14^C] glutamate in the preparations of nerve terminals were conducted, as previously described (Borisova et al., 2016). Briefly, the synaptosomal suspensions (125 μl, 0.5 mg of protein/ml) were pre-incubated at 37°C, for 10 min, and the OMVs (70 μg of protein/ml) were added and incubated for 7 min, following sedimentation (10,000 g, 20 s). The release value was measured in the aliquots of the supernatants (100 μl) and in the pellets by liquid scintillation counting with scintillation cocktail (1.5 ml). The results were expressed in nMol of ʟ-[^14^C] glutamate/mg of protein.

### Animal Ethical Approval

Pregnant ICR mice (body weight 19–23 g) and Wistar rats (body weight 100–120 g) were maintained in special animal facilities at the Institute of Molecular Biology and Genetics and the Palladin Institute of Biochemistry of NAS in Ukraine, respectively. Animals were housed in quiet, temperature-controlled rooms (22–23°C) with a 12 h light:12 h dark cycle (lights on between 08:00 and 20:00 h). The animals were provided with purified water and dry food pellets *ad libitum*. The experimental procedures were carried out according to the standard ethical guidelines (European Community Guidelines on the Care and Use of Laboratory Animals 86/609/EEC) and approved by Institutes’ Ethics Committees.

### Statistical Tests

Results were expressed as mean ± standard deviation (SD) of independent experiments performed in triplicate. The difference between groups was compared by two-tailed Student’s *t*-test. The differences were considered to be significant when *p* ≤ 0.05.

## Results

### Simulated Martian Stressors Promote OMVs With Changed Membranes

Cellulose-synthesizing *K. intermedius* constitutes more than 90% of prokaryotic microbes of the kombucha community (Podolich et al., 2019), and it is a carrier of LPS without O-antigen–endotoxin–integrated into the outer membrane (Barja et al., 2016). Previously, *K. intermedius* were re-isolated from the returned and reactivated space-exposed samples and identified using the 16S rDNA as a phylogenetic marker (Podolich et al., 2019). These strains were used in this study.

**Figure 1:**
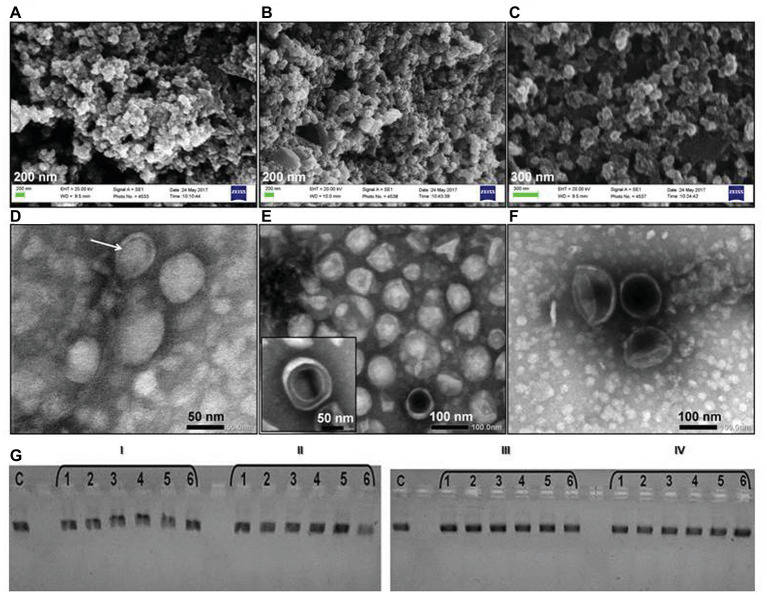
Outer membrane vesicles (OMVs) of the key kombucha microbial community (KMC)-member *Komagataeibacter intermedius* recovered from the KMC samples exposed to the space/Mars-like stressors outside the International Space Station (ISS) and their interaction with a linear plasmid DNA. Scanning electron micrographs of OMVs of reference *K. intermedius* IMBG180 **(A)**; OMVs of *K. intermedius* IMBG185 from KMC exposed at the top level of the carrier on the exposure platform (tKMC) **(B)**; OMVs of *K. intermedius* IMBG183 from KMC exposed at the bottom level of the carrier on the exposure platform (bKMC) **(C)**; transmission electron micrographs of OMVs with one‐ and double-bilayer membranes (indicated with an arrow) derived from initial reference *K. intermedius* IMBG180 **(D)**; IMBG185, in the inset, outer-inner membrane vesicle is shown **(E)**; IMBG183 **(F)**. Scale bars are 50–100 nm. **(G)** Effect of interaction of the *Eco*RI-linearized plasmid pTZ19R (Fermentas, Lithuania) with OMVs of *K. intermedius* from the initial kombucha microbial culture (KMC) (I) and KMCs exposed to Mars-like conditions, in LEO: a top level (II), a bottom level (III), and a middle level (IV). Lane C is the reference *Eco*RI-linearized plasmid pTZ19R. Lanes 1–6 are various concentrations of OMVs (1.25–0.25 mg ml^−1^).

Bacterial OMV samples analyzed by DLS displayed larger average sizes (IMBG185: 70.31 ± 0.5 nm; IMBG184: 78.33 ± 1.3 nm; IMBG183: 66.41 ± 0.2 nm; Supplementary Figure S2), compared to OMVs from ground-based bacteria (IMBG180: 65.81 ± 0.3 nm). The DLS data were in agreement with the data obtained from the SEM and TEM micrographs of OMVs (Figures 1A–F). Furthermore, vesicles originating from the isolates of *K. intermedius* had partially deformed membranes as shown on both the scanning electron microscopy and TEM micrographs in Figures 1B,E.

Gram-negative bacterium *K. intermedius* IMBG180 releases conventional one-bilayer OMVs and, to a lesser extent, outer-inner membrane vesicles (characterized by the protrusion of both outer and plasma membranes; Figure 1D). The same pattern was visible in vesicles produced by *K. intermedius* strains recovered from KMCs exposed to space/Mars-like stressors (inset of Figure 1E). In OMVs, only the periplasm is entrapped while in more complex vesicles, with a double-bilayer structure, cytoplasmic content can also be entrapped, including such cellular components as DNA, RNAs, enzymes, and other metabolites. Exogenous DNA was detected in all OMV preparations, including OMVs from a reference strain, by staining with the fluorogenic DNA-binding dye DAPI (data not shown). When intact OMVs were treated with DNAse I, the DNA in the OMV preparations was digested by the enzyme. It indicates that DNA molecules were located on the vesicle surface. Although the presence of a small size RNA (42 nt) was detected with the Agilent 2100 Bioanalyzer (Thermo Scientific; data not shown), the isolation of RNA from OMVs after the treatment with RNase A did not yield detectable material. Post-stress *K. intermedius* OMVs had a negative charge; however, their zeta potential changed from −5.5 (OMVs/iKMC) to −1.5 mV (OMVs/tKMC).

### Electrophoresis Mobility Shift Assay May Show Membrane Surface Alterations

Changes in the post-stress *K. intermedius* OMVs’ membrane surface charge can be reflected in the OMV interactions with biopolymers, e.g., polynucleotides. Electrophoresis mobility shift assay (EMSA) is a rapid and sensitive method to detect nucleic acid complexation with polymers, e.g., membrane proteins (Garner and Revzin, 1986). The electrophoretic patterns of the linearized plasmid pTZ19R DNA showed the concentration-dependent formation of molecular complexes with OMVs from non-exposed strain, which slowed their movement in the gel, and did not exhibit the mobility shifts after incubation with vesicles originated from *K. intermedius* isolated from the exposed KMCs (Figure 1G).

### Mars-like Stressors Alter Lipid Structures in OMVs

**Figure 2:**
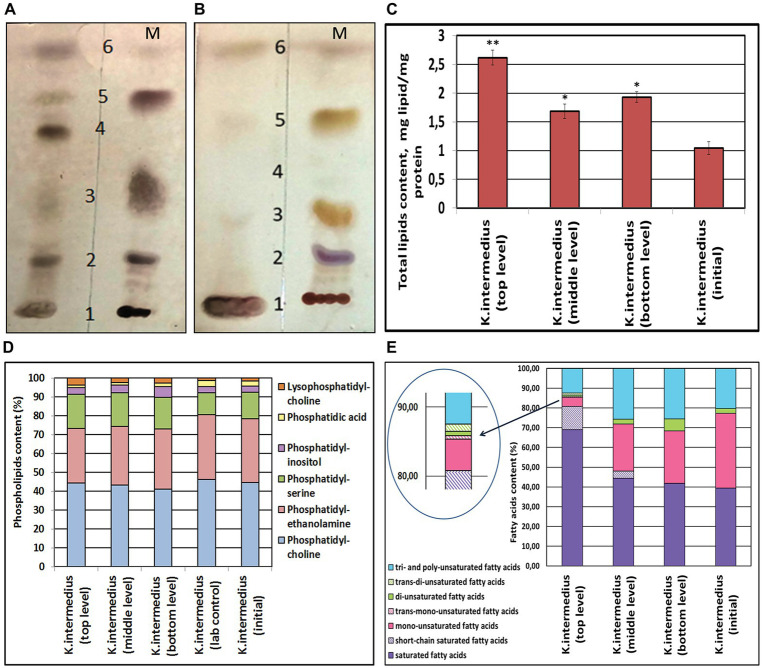
Classes of lipids of OMVs of bacterial monocultures *Komagataeibacter intermedius* **(A,B)**; alterations in the lipid content in OMVs of *K. intermedius* strains recovered from post-spaceflight kombucha microbial cultures (KMC) **(C,D)**. **(A)** Representative thin layer chromatography separation of lipids isolated from OMVs of *K. intermedius* IMBG185 recovered from the top level of the exposure platform (on the left); on the right–lipid markers (M): 1 – phospholipids (PLs); 2 – sterols; 3 – 1,3-diglycerides; 4 – free fatty acids; 5 – triglycerides; 6 – sterol ethers. **(B)** Lipids of OMVs of initial *K. intermedius* IMBG180 (on the left); on the right–markers (M). **(C)** Content of total lipids in OMVs of *K. intermedius* strains of post-spaceflight KMCs. **(D)** PLs of OMVs of *K. intermedius* strains of post-spaceflight KMCs. **(E)** Content of fatty acids in OMVs of *K. intermedius* strains of post-spaceflight KMCs. Three replicates were used in the experiments on lipid content measurements. Data were shown as mean ± SD (*n* = 3). ^*^p ≤ 0.05; ^**^p ≤ 0.01.

As OMVs are derived from outer membranes *via* budding, they carry information about their composition. By studying the membrane lipid profiles in post-stress *K. intermedius* OMVs and comparing them with the profile characterizing non-extreme conditions, we aimed to determine the response of bacterial lipids to simulated Martian stressors. Compositional analysis of membrane lipids revealed that vesicles of all *K. intermedius* strains consisted mostly of the following classes of lipid molecules: PLs, triglycerides, FAs, and sterols. Similarly, across all the strains, several minor lipid components of the OMVs such as 1,3-diglycerides, sterol ethers, and monoglycerides were also revealed (Figures 2A,B).

The total yield of lipids (in milligram of protein) in the OMVs of *K. intermedius* re-isolated from all the tKMC, bKMC, and mKMC space samples increased, compared with the reference (Figure 2C). After the spaceflight experiment, the relative content of zwitterionic phosphatidylethanolamine, as well as anionic PL phosphatidic acid decreased while other anionic PLs like phosphatidylserine and lysine-phosphatidylcholine increased (Figure 2D and Supplementary Table S1), probably producing a change in surface properties of outer membranes, including their charge.

The free FA content of OMVs isolated from the *K. intermedius*/tKMC increased significantly, compared to the untreated control (Figure 2A). The quantity of total saturated FAs had a 1.2–2 fold (*p* < 0.05) increase while unsaturated FAs (mainly monounsaturated) decreased 7.5 fold (*p* < 0.05) in OMVs from *K. intermedius*/tKMC, in contrast to wild type *K. intermedius* OMVs/iKMC. The amount of unsaturated FAs–polyenes–was found to have increased by 1.2–1.3 fold (*p* < 0.05) in OMVs of re-isolates from mKMC and bKMC as compared to preparations of OMVs from the reference strain (Figure 2E and Supplementary Table S2). At the same time, it was established that the UV-illumination caused the appearance of short-chain saturated FAs (С6:0–С11:0) in the OMVs/tKMC and OMVs/mKMC and trans FAs – elaidic (18:1w9) and linolelaidic (18:2w6) – in the OMVs/tKMC.

### Functionality of OMVs After Exposure of *K. intermedius* to Mars-like Stressors

#### A Weaker Membrane Proton Translocation

**Figure 3:**
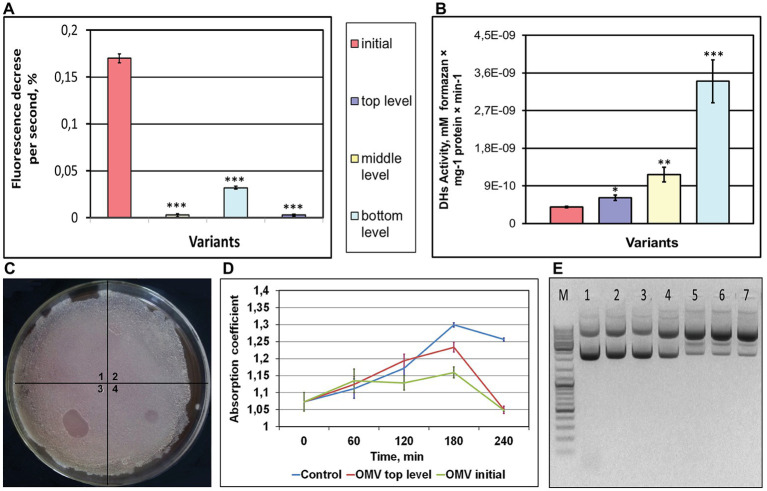
Functionality of OMVs of *K. intermedius*, isolated from post-flight KMCs **(A,B,D,E)** and extracellular vesicles (EVs) isolated from the post-flight KMC samples **(C)**. **(A)** OMVs acidification measured using a pH-sensitive fluorescent dye acridine orange. Typical recording of acidification of OMVs from *K. intermedius* isolated from initial KMC and KMCs located at the top, middle, and bottom levels of a carrier mounted outside the ISS. **(B)** Dehydrogenase activity of OMVs of *K. intermedius* re-isolated from different KMCs. Data were shown as mean ± SD (*n* = 3), ^*^p ≤ 0.05; ^**^p ≤ 0.01; ^***^p ≤ 0.001. **(C)** The image of *Bacillus subtilis* lawn spotted with EVs. Clear zones are seen in spots of EVs from initial KMC (4) and EVs from bottom-located KMC (2). No visible changes seen after spotting with EVs of top‐ (1) and middle-located KMC (3). **(D)** Inhibitory activity of OMVs from *K. intermedius* in regard to *B. subtilis*. **(E)** Electrophoregram of plasmid pTZ19R after incubation with OMVs of *K. intermedius*. M–DNA ladder (ThermoFisher Scientific GeneRuler DNA Ladder Mix). 1 – control DNA (without incubation); 2 – control DNA (incubation, 37°С); 3 – control DNA (+PBS, incubation, 37°С); 4 – OMVs of *K. intermedius* from initial KMC; 5–7 – OMVs of *K. intermedius* from bottom-, middle-, and top-located KMCs, incubated with DNA at 37°С.

Structure deviations in cell/OMV membranes may lead to changes in membrane permeability, and we assessed a proton gradient formation in OMVs of post-flight *K. intermedius* strains. A pH-sensitive fluorescent dye, acridine orange, is commonly used to detect acidification in the whole cells, isolated lysosomes, endocytotic granules, and synaptic vesicles (Zoccarato et al., 1999; Borisova, 2014). Acridine orange is easily permeable *via* the membranes as the unprotonated amine, and thereafter, it converts itself to the protonated form and is concentrated in the acidic compartments, where this dye dimerizes, changing the optical features (Zoccarato et al., 1999). As shown in Figure 3A, an addition of acridine orange dye to OMVs/*K. intermedius*/iKMC caused a partial quenching of the fluorescence signal due to an accumulation of the dye inside of the vesicles that indicated a definite vesicle acidification. In contrast, a treatment of OMVs/*K. intermedius*/tKMCs with acridine orange did not change the fluorescence intensity of the dye. OMVs of *K. intermedius* from other locations – medium and bottom levels – exhibited some capability to form proton gradient; however, it was weaker compared to *K. intermedius* OMVs from initial KMC.

### Deviations in Enzymatic Activities Associated With Membrane Vesicles in Post-flight Microorganisms

Membrane leaflet is a harbor of bioactive molecules, including enzymes, and alterations in membrane lipid composition may lead to changes in its activities or its deprivation. We selected a set of membrane-associated enzymatic activities for OMVs/EVs (dehydrogenases, DNAse, hydrolyses) to study their activities associated with vesicles from exposed microorganisms.

#### Increased Dehydrogenase(s) Activity

Dehydrogenases are enzymes belonging to a group of oxidoreductases that oxidize the substrate by transferring protons and electrons through a chain of intermediate electron carriers [usually, Flavin adenine dinucleotide (FAD)‐ or pyrroloquinoline quinone (PQQ)-dependent in Gram-negative bacteria] to a final electron acceptor (Adachi et al., 2007). These activities have increased in all OMVs from post-exposed strains of *K. intermedius*: 1.6-fold (from tKMC), 3-fold (from mKMC), and 8.6-fold (from bKMC), when compared to a control sample from wt *K. intermedius* (Figure 3B).

#### Changed Antimicrobial Activity Against Gram-Positive Bacteria

Antimicrobial compounds and bacteriolytic enzymes, including autolysins carried by bacterial OMVs, are suggested to exert an inhibitory or killing effect on co-existing bacteria or fungi (Vasilyeva et al., 2013; Schulz et al., 2018). EVs from initial KMC had a weak lytic action on the *B. subtilis* lawn in the form of a translucent lysis zone (Figure 3C). A stronger lytic effect was seen as a clear visible zone of lysis at the site of application by EVs/bKMC. No clear spots were produced by EVs from tKMC and mKMC, or even a negative control. Nonetheless, in the case of Gram-negative bacterial species *E. coli* and *P. aeruginosa*, which have different membrane structures as compared to Gram-positive bacilli, the results were the opposite: EVs of KMCs supported growth of these cultures (data not shown) and probably were used as nutrients. The antimicrobial activity in relation to Gram-positive bacilli was less expressed in *K. intermedius* OMVs from exposed bacteria compared to EVs/bKMC and the wt OMVs (Figure 3D).

#### Higher Level of Deoxyribonuclease Activity

Nucleases perform different roles to fit bacterial lifestyle (Al-Wahaibi et al., 2019; Pressler et al., 2019). Extracellular nucleases are known as enzymes to accommodate invading bacteria in hosts *via* the formation of a set of diverse oligonucleotides with immunomodulatory properties (Takahashi et al., 2006) or help evade the innate immune response (Jhelum et al., 2018). Single-stranded specific deoxyribonuclease activity was detected on a formation of open-circular and linear plasmid DNA forms from a supercoiled form of pTZ19R by *K. intermedius* OMVs (Figure 3E, *wells* 5–7). Conversely, control OMVs had lower activity and formed only an open-circular form of the plasmid (Figure 3E, *well* 4).

### Martian Stressors Practically Do Not Alter Cytotoxicity and Endotoxicity of Extracellular Vesicles

**Figure 4:**
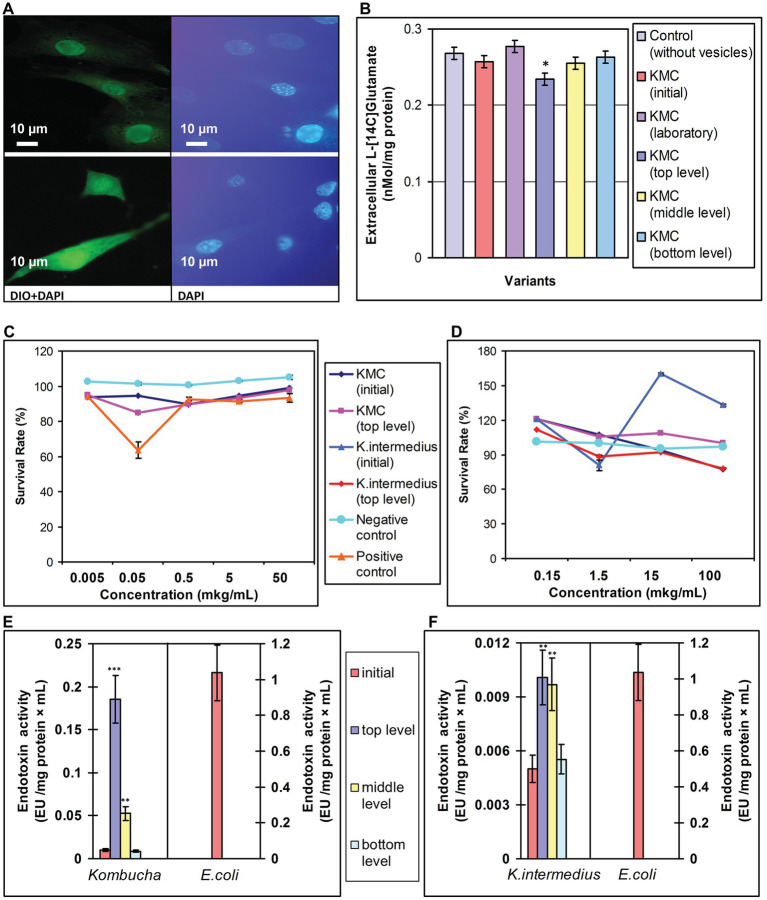
A biosafety assessment of EVs after exposure of kombucha multimicrobial culture (KMC) under space/Mars-like stressors outboard the ISS and OMVs of *K. intermedius*, isolated from post-flight KMCs. **(A)** A micrograph of OMVs stained with the lipophilic dye DiO visible in murine fibroblasts. Fibroblast nuclei were stained with DAPI. (1, 2) – OMVs of *K. intermedius* from initial KMC; (3, 4) – OMVs of *K. intermedius* from top-located KMC (tKMC). Scale bar, 10 μm. **(B)** The environment level of ʟ-[^14^C] glutamate in the preparations of rat brain nerve terminals after co-cultivation with EVs from post-flight KMCs. **(C)** Survival rate of murine embryo fibroblasts after co-cultivation with different concentrations of OMVs from *K. intermedius* isolated from tKMC. **(D)** Survival rate of colorectal carcinoma cells COLO 205 after co-cultivation with different concentrations of OMVs/*K. intermedius*, isolated from KMCs exposed outboard the ISS. **(E)** Endotoxin activity of EVs/KMCs detected with the *Limulus* amebocyte lysate (LAL) assay compared to standard endotoxin activity from *Escherichia coli*. **(F)** Endotoxin activity of OMVs/*K. intermedius* detected with the LAL assay. Data were shown as mean ± SD (*n* = 3), ^*^p ≤ 0.05; ^**^p ≤ 0.01; ^***^p ≤ 0.001.

As exposed *Komagataeibacter* possess LPS in their outer membrane, known as endotoxin, and as both this bacteria and KMC are promising probiotics, we examined their safety after a spaceflight, using vesicles as their representatives. First, OMVs were labeled with lipophilic dye DiO and introduced into MEF to show the vesicles interaction with murine cells. The cell interior of MEF showed the fluorescence of DiO labeled OMVs of both wt and space-exposed (UVC-illuminated) *K. intermedius* (Figure 4A, panels 1 and 3). Exposure of MEF cells to total vesicle preparation isolated just after revival of returned tKMC had a small inhibitory effect (15%) on the cell growth at concentration of 50 ng ml^−1^ (Figure 4C) compared to vesicles of iKMC. Nevertheless, this effect did not reach a threshold level (50%) and did not cause cytotoxicity in MEF. Co-incubation of the OMVs with cell culture COLO 205 did not exhibit cytotoxicity of the OMVs in both spaceflight-related and control OMVs (Figure 4D).

EVs isolated from iKMC, representing the LPS-bearing fraction of Gram-negative bacteria (including *K. intermedius*), displayed 50-fold lower endotoxin activity level in the LAL assay compared to *E. coli*, as an endotoxin standard (Figure 4E). Levels of endotoxin activity in metavesicleomes from tKMC and mKMC were 18.4‐ and 5-fold higher, respectively, compared to the iKMC. Nonetheless, their levels were 21‐ and 6-fold lower, compared to *E. coli.* Endotoxin activity of OMVs of bacteria isolated from tKMC and mKMC exhibited a 2-fold increase compared to the OMVs from *K. intermedius*/iKMC, and it was less than 0.01% of the *E. coli* activity (Figure 4F). The level of endotoxin activity of OMVs/*K. intermedius*/tKMC was practically the same as in vesicles of the iKMC.

### Martian Stressors Do Not Cause Neuromodulation by Extracellular Vesicles From KMCs

The ambient glutamate level between episodes of exocytosis is a crucial characteristic of synaptic neurotransmission and depends on numerous individual presynaptic parameters (Borisova, 2019). The total nanovesicle pool of revived post-flight KMCs did not increase the ambient level of ʟ-[^14^C] glutamate in the synaptosome suspensions in the rat brain nerve terminals, i.e., it did not acquire neuromodulation. The EVs of tKMC even decreased the ʟ-[^14^C] glutamate content, but EVs from KMC specimens, exposed at the middle and bottom levels, did not differ from the EVs of iKMC (Figure 4B).

## Discussion

Within the BIOMEX project, different extraterrestrial stressors subjected to different sample carrier layers had a distinct mode of action, and some of them were lethal to KMC-members, resulting in changes in the microbial community structure (Podolich et al., 2019). A key community member, the Gram-negative bacterium *K. intermedius*, has survived under harsh Mars-like radiation, atmosphere, and low gravity within the returned KMC samples. OMVs mirrored, to some extent, changes in their outer membrane structure, as well as membrane-associated activities of the *K. intermedius* re-isolates. An increase in both *K. intermedius* OMVs size and their number resulted from microbial adaptation to extraterrestrial stressors, apparently *via* cellular envelope remodeling, which is known from other Gram-negative bacteria exposed to stressful environmental or induced conditions (Sabra et al., 2003; Gerritzen et al., 2019; Klimentova et al., 2019). The increase in envelope thickness and production rate of OMVs with respect to the ground-based reference was previously demonstrated in the spaceflight samples of *E. coli* under microgravity (Zea et al., 2017).

### Long-Duration Exposure of KMC Samples on the ISS Caused a Modification of Lipid Composition of the Cellular Membrane, a Change of Surface Properties, and Membrane Permeability

The core lipids of *K. intermedius*, which serve as the framework for fully mature membrane lipids, are PLs, triglycerides, FAs, and sterols. Typical membrane parameters adversely affected by environmental challenges are permeability and fluidity, which, in turn, affect the function and mobility of membrane proteins, diffusion of nutrients, and energy sources (Rowlett et al., 2017; Siliakus et al., 2017; Jasim et al., 2018). In our study, the most striking changes were recorded in the membranes of OMVs from the UV-exposed *K. intermedius*, compared to the OMVs from *K. intermedius*/iKMC, and this correlated with increased synthesis of lipids, especially, PLs, free FAs, and sterols, which could cause alteration in membrane fluidity. Shortening of FA chain lengths occurred, probably by degrading the unfavorable membrane PLs that retained adequate membrane fluidity during the bacterial growth. Detected imbalance in saturated and unsaturated FAs and increased membrane condensation in cells/OMVs of *K. intermedius*/tKMC is an adaptive response to stressors that allow bacteria to limit metabolism, save energy, and survive under harsh conditions of Mars-like UV, simulated pressure, and atmosphere. Deformed membranes of the OMVs (tKMC), as shown in both the scanning electron microscopy and TEM micrographs, can be explained by membrane saturation leading to its rigidity. Additional strategies were also adopted by cells, such as maintenance of a changed membrane potential and development of a chemiosmotic barrier to protons. The polar PL head groups could also play a significant role in the maintenance of membrane fluidity and permeability. The membrane PLs diversity allows for different non-covalent forces, i.e., van der Waals, electrostatic, solvation (hydration and hydrophobic), steric, and entropic. Changes in the PL pattern led to alteration of the membrane surface properties, including charge, and this was reflected in the OMV interactions with biopolymers. For instance, modified membranes of vesicles could lose the capability to interact and form complexes with polynucleotides, in contrast to the reference OMVs, which interacted with plasmid DNA, resulting in the formation of supramolecular complexes or aggregates. Our data on the membrane lipid composition alterations are consistent with the previously reported membrane lipid modifications, including PL content, occurring under other stressful conditions in closely related acetic acid bacteria (Trcˇek et al., 2007; Ogawa et al., 2010) or other bacteria (Pei et al., 2019).

The capability to form proton gradient of post-flight OMVs was weaker, as compared to the OMVs of initial untreated strain. The bacterial membrane controls the traffic of various molecules into and out of the cell and also participates in signal transduction and chemical processing of incoming molecular species. The outer membrane contains porins involved in the influx of various compounds in bacteria (Masi et al., 2019), and membrane H^+^-ATPase, which performs secondary active transport processes across the membrane, energizing the membrane and forming an electrochemical gradient of protons (Turina et al., 2006). *K. intermedius* OMVs from KMC, exposed at upper unprotected level of the sample carrier, had very low capacity to build the proton gradient. It should be noted that UV can act as a trigger for modulation of membrane structure modification, leading to either loss of implicated proteins or their suppression (Ghorbal et al., 2019). To sum up, Mars-like illumination, gravity, and atmosphere influenced outer membrane alterations, and UV, as a stressful factor, was the most pronounced for *K. intermedius*. Usually, UV is detrimental for microorganisms and even fatal, especially when UV-C induces bacterial death by targeting DNA (Dai et al., 2012). Bacterial exposure to sublethal UV doses leads to membrane PL bilayer rearrangement and pore formation (Smith et al., 2009), inducing changes in the amounts and composition of FAs (Khefacha et al., 2015) and affecting LPSs (Chourabi et al., 2017). These modifications caused by UV lead to altered membrane permeability (Santos et al., 2013). Under stressful conditions, bacterial adaptation, regarded as a stress response to the physicochemical change, occurs and aids in the survival of bacterial cell populations *via* physiological membrane adaptations (Siliakus et al., 2017).

Most of the structural changes occur in the membranes of unprotected microorganisms exposed to UV similar to Martian surface, translated in proton translocation, and interacted with synaptosomes. Other complex stressors affected exposed bacteria that were protected from UV. For example, the appearance of short-chain saturated FAs in membranes of bacteria/OMVs from middle level of exposure has been recorded. Comparative genomics of wild-type and post-stress *K. intermedius* revealed a reduction of genome in the UV-irradiated returned bacteria (manuscript in preparation). We hypothesize, that such changes may be related to multiple deletions.

### The Changes in Cell Membranes Correlated With the Altered Functional Membrane-Associated Capabilities of OMVs

In this study, we observed increased DNAse activity in OMVs of post-stress *K. imtermedius* from all levels of exposure (top, middle, and bottom) compared to ground reference. This phenomenon can be attributed to modifications in membrane structure, more specifically, in PL pattern, and, consequently, membrane charge. As OMVs of reference *K. intermedius* probably may change the topological form of the plasmid linear DNA, e.g., as under the influence of topoisomerase, leading to knotting or catenation of DNA (Portugal and Rodríguez-Campos, 1996), OMVs of post-stress bacteria did not change the spatial architecture of linear DNA and had target DNA more accessible. Enhanced DNAse activities observed in OMVs from post-spaceflight re-isolates correlated with a poor biofilm production in parental bacteria, when the activity of such nucleases was demonstrated to balance the extent of biofilm formation, and this is congruent with the previously published results by Sharma and Singh (2018). Nucleases also contribute to the lower bacterial transformation capability of different bacteria, preventing horizontal gene transfer (Binnenkade et al., 2018; Jhelum et al., 2018). The nuclease-associated OMVs of the dominant KMC *K. intermedius* can be represented as safeguards, keeping the essential genome stability in this microecosystem. This is the first step toward the understanding of enzymatic properties of DNAses associated with OMVs, affecting *K. intermedius* survival, genome stability, and cellulose-synthetic fitness in the planktonic and pellicle environment of kombucha multi-species community.

We also observed increased dehydrogenase(s) activity in the order top > middle > bottom of reference OMV samples. In acetic acid bacteria of the *Komagataeibacter* genus, membrane-bound dehydrogenases are mainly represented by PQQ-dependent alcohol dehydrogenase, and the glucose dehydrogenase that contains PQQ as the prosthetic group (Matsutani and Yakushi, 2018). PQQ-dependent dehydrogenases catalyze the oxidation of non-phosphorylated substrates and can be beneficial for detoxifying cellular toxic compounds. In some organisms, they are induced under energy stress, providing an auxiliary energy generation pathway (Tribelli et al., 2015). In other organisms, they play a role in maintaining the transmembrane proton gradient (Liu et al., 2018). In our study, the increase in total DH-activities of post-spaceflight *K. intermedius* OMVs probably mirrors the compensatory mechanism of impaired proton gradient formation: the less proton gradient is, the higher membrane-associated DH-activities are.

Nanovesicle preparations derived from wild ecotype of KMC showed a killing activity toward *B. subtilis*, in a context of known KMC antimicrobial properties (Chakravorty et al., 2019), probably due to the hydrolytic enzyme(s), destroying the cell wall of Gram-positive bacterium. On the other hand, total vesicles from UV-illuminated tKMC lost this activity. Notably, EVs from “dark” bKMC did not change antimicrobial activity toward bacilli. OMVs isolated from *K. intermedius* did not exhibit a lytic activity; however, inhibited the growth of bacilli while OMVs isolated from *K. intermedius*/tKMC exhibited lower inhibitory activity toward *B. subtilis*.

In order to summarize the results on enzymatic activities associated with *K. intermedius* OMVs, we suppose that, as long as membrane-associated enzymes used in this study belong to the integral outer membrane protein types (e.g., DNAse belong to the β-barrel superfamily of phospholipase D; Nelson and Frohman, 2015) and penetrate the peripheral regions of the lipid bilayer, an activation of their biological activity depends on the impact of stressors that can perturb the membrane. Alterations in cell membrane result in the promotion of conformational changes within the enzyme structural domains and in their activity (Johnson and Cornell, 1999). Alternatively, changes in the outer membrane structure may prevent enzyme integration and result in a loss of appropriate function.

### Alterations in Microbial Membranes Inevitably Lead to a Change in the Mode of Communications With Eukaryotic Cells and Its Nanostructures

Changes in the *K. intermedius* OMV membrane structure were vital for its communications not only with alien bacteria, but also with mammalian cell cultures. In this study, internalized OMVs interacted with MEF cells and human colorectal carcinoma cells but these communications did not result in acquisition of cytotoxicity. Despite occurring at low concentrations, the OMVs inhibited both murine and human cell proliferation. The metavesicleome of post-flight KMCs did not increase the ambient level of ʟ-[^14^C] glutamate in the synaptosome suspensions in the rat brain nerve terminals, i.e., it did not acquire neuromodulation, and the vesicles of tKMC even decreased the ʟ-[^14^C] glutamate content. Biological activity of the post-flight *K. intermedius* OMV-associated LPS was slightly increased; however, it was almost identical to the wt KMC/EVs LPS-activity. To sum up, we can conclude that, despite alterations in membranes after the impact of extraterrestrial and simulated stressors outside the ISS, *K. intermedius* OMVs did not acquire endotoxicity, cytotoxicity, and neuromodulation. Therefore, OMVs, originating from carefully selected nonpathogenic Gram-negative bacteria, can be considered as potential candidates in the design of postbiotics or edible mucosal vaccines for *in situ* production in extreme environments, besides being promising delivery vectors for applications in Astromedicine.

## Data Availability Statement

All datasets generated for this study are included in the article/Supplementary Material.

## Ethics Statement

This study was carried out in accordance with the standard ethical guidelines (European Community Guidelines on the Care and Use of Laboratory Animals 86/609/EEC) and approved by the Ethics Committees of the Institute of Molecular Biology and Genetics and the Palladin Institute of Biochemistry of NAS in Ukraine.

## Author Contributions

OP, J-PV, and NK conceived and designed the experiments. NK, GZ, DB, and PG were responsible for drafting the article. IZ, OK, IO, OR, LP, LZ, TH, MG, VK, MS, and SS were involved in performing the experiments and TB, HK, DB, AG-N, VA, MK, and NK analyzed the data. All authors reviewed and approved the final version of the manuscript.

## Conflict of Interest

MK, VK, and MS were employed by NanoMedTech LLC, Kyiv.

The remaining authors declare that the research was conducted in the absence of any commercial or financial relationships that could be construed as a potential conflict of interest.

## Acknowledgments

Authors thank Ms. Rosa Suleimanova, Dr. V.M. Klimashevsky, Ms. Natalia Andryushyna, and Ms. Natalia Melnychuk for their excellent technical assistance.

## Supplementary Material

The Supplementary Material for this article can be found online at: https://www.frontiersin.org/articles/10.3389/fmicb.2020.01268/full#supplementary-material.
