# Supplementary material for: Fitness of Outer Membrane Vesicles From Komagataeibacter intermedius Is Altered Under the Impact of Simulated Mars-like Stressors Outside the International Space Station
Source: Front Microbiol. 2020 Jun 26;11:1268. doi: 10.3389/fmicb.2020.01268 (PMC7333525; doi:10.3389/fmicb.2020.01268)
Supplement: Supplementary file 1 [file Data_Sheet_1.PDF]

SUPPLEMENTARY INFORMATION

Fitness of outer membrane vesicles from *Komagataeibacter intermedius* is changed under the impact of simulated Mars-like stressors outside the International Space Station

Olga Podolich, Olga Kukharenko, Iryna Zaets, Iryna Orlovska, Larysa Palchykovska, Leonid Zaika, Serhii Sysoliatin, Ganna Zubova, Oleg Reva, Maxym Galkin, Tetyana Horid'ko, Halyna Kosiakova, Tatiana Borisova, Volodymyr Kravchenko, Mykola Skoryk, Maxym Kremenskoy, Preetam Ghosh, Debmalya Barh, Aristóteles Góes-Neto, Vasco Azevedo, Jean-Pierre de Vera, Natalia Kozyrovska

Figure S1. The view and location of kombucha bio-mineral samples integrated into a carrier C2 of tray 2 of the BIOMEX experiment. A, the carrier C2 in tray 2 has a three-level architecture (top, middle, and bottom levels), where each level was hosting four kombucha samples (indicated with red circle) and maintained under a simulated Mars atmosphere (95.55 % CO_2, 2.70 % N_2, 1.60 % Ar, 0.15 % O_2, ~ 370 ppm H_2O) and a pressure of 980 Pa. The top level samples were exposed to a solar UV flux cut off by optical filters to wavelengths of > 200 nm as prevalent on the Martian surface. The middle- and bottom-located samples were UV-protected. B, EXPOSE-R2 flight trays with all experiments integrated is outboard the International Space Station, in astronaut's hands, before a mounting on a platform. Credit: ESA, Roscosmos.

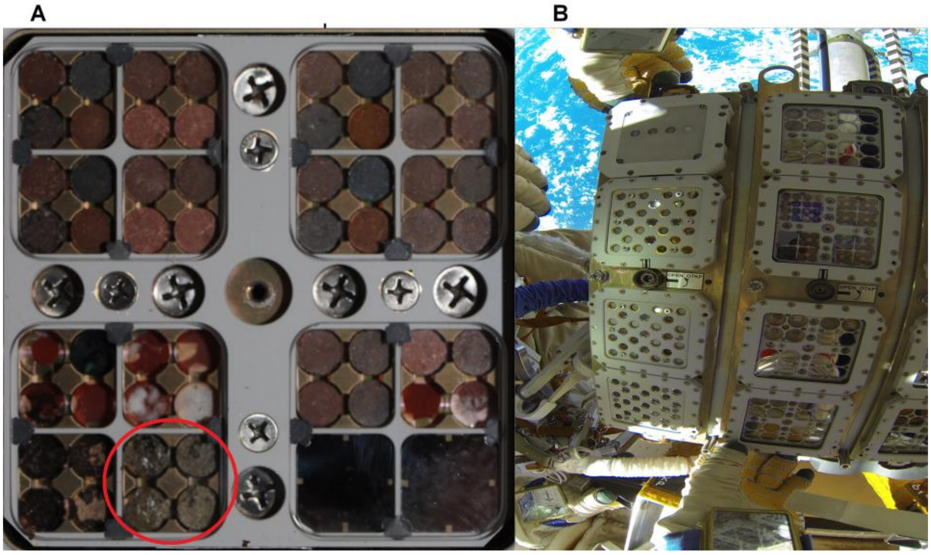

Figure S2. Size statistics of outer-membrane vesicles produced by *Komagataeibacter intermedius* IMBG185 (a top-level location) (A); *K. intermedius* IMBG184 (a medium-level location) (B); *K. intermedius* IMBG184 (a bottom-level location) (C); *K. intermedius* IMBG180 (from initial KMC) (D), Zeta Sizer Nano S (Malvern Instrumental Ltd, 2008).

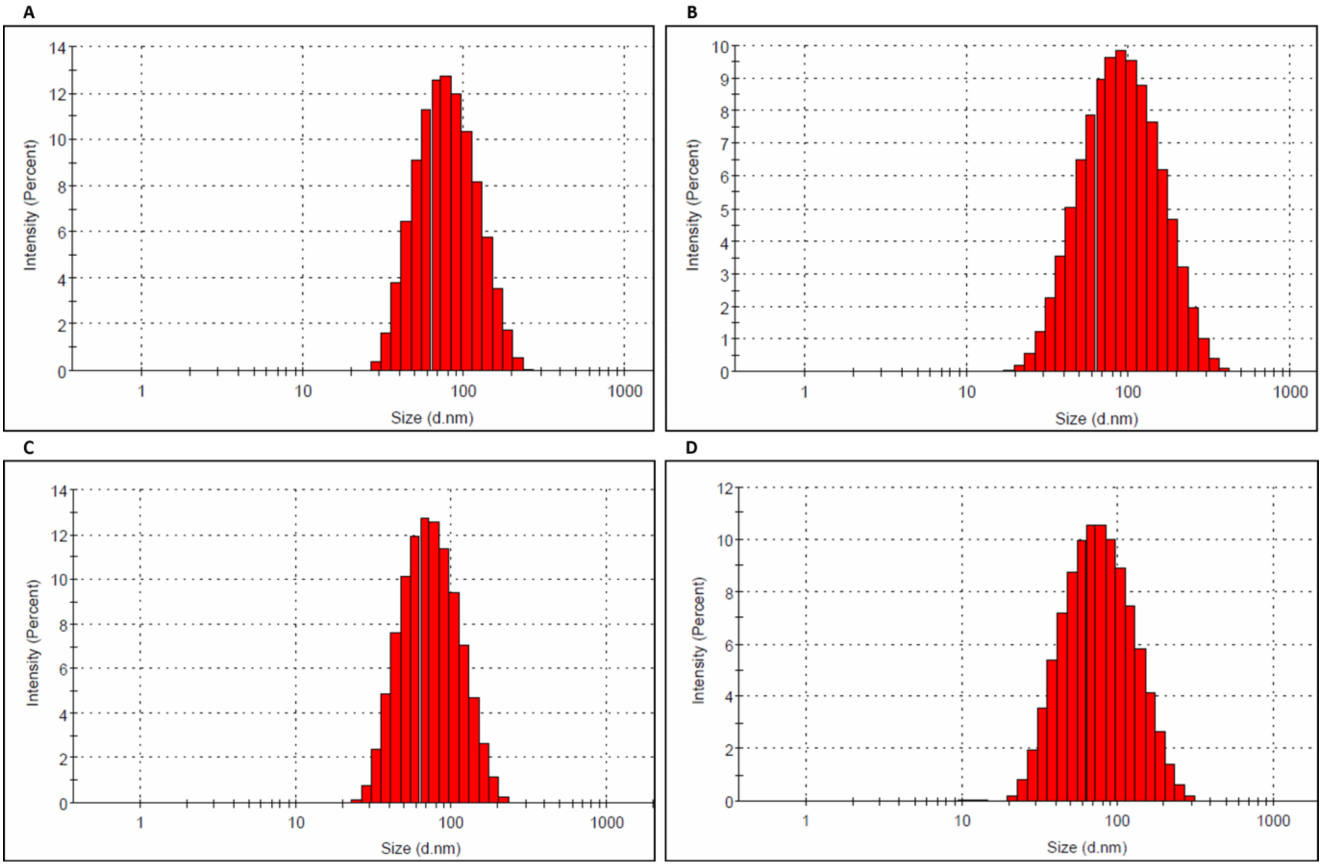

Table S1. Phospholipids content in outer-membrane vesicles of bacterial monoculture *Komagataeibacter intermedius*, %

**Phospholipids content**	**K.intermedius (top level)**	**K.intermedius (middle level)**	**K.intermedius (bottom level)**	**K.intermedius (lab control)**	**K.intermedius (initial)**
Phosphatidyl-choline	44.38±0.88	43.31±0.68 *	41.28±0.60 *	46.16±0.75	44.72±0.52
Phosphatidyl-ethanolamine	29.00±0.90 *	30.94±1.01 *	31.72±1.32	34.45±1.39	33.79±0.98
Phosphatidyl-serine	18.12±0.99 *	17.83±0.95 *	16.67±1.46 *	11.54±1.31	13.89±0.91
Phosphatidyl-inositol	3.52±1.13	4.14±0.91	5.73±1.55	3.23±0.98	3.38±1.31
Phosphatidic acid	1.25±0.50 *	1.44±0.67 *	1.85±0.84	3.24±0.54	2.51±0.35
Lysophosphatidyl-choline	3.75±0.41 *	2.34±0.43 *	2.75±0.63 *	1.38±0.83	1.71±0.15

Data were shown as mean ± SD (n = 3)
**P≤0.05*

Table S2. Fatty acids content in outer-membrane vesicles of bacterial monoculture *Komagataeibacter intermedius*, %

**Fatty acids content**	**K.intermedius (top level)**	**K.intermedius (middle level)**	**K.intermedius (bottom level)**	**K.intermedius (initial)**
saturated fatty acids	68.95±2.03 **	44.32±1.45*	41.84±1.67	39.4±1.14
short-chain saturated fatty acids	11.89±0.91 ***	3.65±0.49 **	0.00	0.00
mono-unsaturated fatty acids	4.54±0.65 ***	23.98±0.96 **	26.53±1.06 **	37.88±1.10
trans-mono-unsaturated fatty acids	0.5±0.15 *	0.00	0.00	0.00
di-unsaturated fatty acids	0.61±0.14 *	2.4±0.53	6.12±0.93 *	2.52±0.88
trans-di-unsaturated fatty acids	1.11±0.23 *	0.00	0.00	0.00
tri- and poly-unsaturated fatty acids	12.4±1.34 **	25.65±1.52 *	25.51±0.99 *	20.2±1.12

Data were shown as mean ± SD (n = 3)
**P≤0.05; **P≤0.01; ***P≤0.001*
